# Non-native Speech Learning in Older Adults

**DOI:** 10.3389/fpsyg.2017.00148

**Published:** 2017-02-10

**Authors:** Erin M. Ingvalson, Casandra Nowicki, Audrey Zong, Patrick C. M. Wong

**Affiliations:** ^1^School of Communication Science and Disorders, Florida State University, TallahasseeFL, USA; ^2^Department of Otolaryngology – Head and Neck Surgery, Feinberg School of Medicine, Northwestern University, ChicagoIL, USA; ^3^Roxelyn and Richard Pepper Department of Communication Sciences and Disorders, Northwestern University, EvanstonIL, USA; ^4^Department of Linguistics and Modern Languages, The Chinese University of Hong KongHong Kong, Hong Kong; ^5^CUHK-Utrecht University Joint Centre for Language, Mind, and Brain, The Chinese University of Hong KongHong Kong, Hong Kong

**Keywords:** second language learning, non-native speech perception, working memory, declarative memory, older adults

## Abstract

Though there is an extensive literature investigating the ability of younger adults to learn non-native phonology, including investigations into individual differences in younger adults’ lexical tone learning, very little is known about older adults’ ability to learn non-native phonology, including lexical tone. There are several reasons to suspect that older adults would use different learning mechanisms when learning lexical tone than younger adults, including poorer perception of dynamic pitch, greater reliance on working memory capacity in second language learning, and poorer category learning in older adulthood. The present study examined the relationships among older adults’ baseline sensitivity for pitch patterns, working memory capacity, and declarative memory capacity with their ability to learn to associate tone with lexical meaning. In older adults, baseline pitch pattern sensitivity was not associated with generalization performance. Rather, older adults’ learning performance was best predicted by declarative memory capacity. These data suggest that training paradigms will need to be modified to optimize older adults’ non-native speech sound learning success.

## Introduction

Though there is an extensive literature investigating non-native speech learning in younger adults, very little is known about the non-native speech learning abilities of older adults. One place where this disparity is particularly apparent is in investigations of lexical tone. In younger adults, it has been demonstrated that listeners are able to learn to both perceive and produce lexical tone (Wang et al., 1999, 2003) and that individual variation in learning outcomes can be predicted by individual variation in baseline sensitivity to non-lexical pitch patterns (Wong and Perrachione, 2007; Perrachione et al., 2011). Additionally, it has been shown that listeners with poor baseline sensitivity for non-lexical pitch patterns have improved learning outcomes by reducing the acoustic variability in the training context (Perrachione et al., 2011). Another means of improving learning outcomes for listeners with poor baseline sensitivity for non-lexical pitch patterns is through explicit training on pitch patterns prior to lexical tone learning (Ingvalson et al., 2013).

There are several reasons to believe that older adults may show different learning outcomes, and different learning mechanisms, for lexical tone compared to younger adults. The first of these is the difficulty older adults, even those who have normal hearing, have perceiving dynamic pitch (Shen et al., 2016). Assessed on their ability to identify rising or falling pitch contours, older adults’ identification curves were shallower than younger adults’ identification curves, indicating that the younger adults were better at the identification task. Younger adults also out-performed older adults when discriminating dynamic pitch. Within the younger adult group, those listeners who had musical training had steeper curves than did the listeners without musical training; no such musicianship advantage was seen for the older adults. Given that dynamic pitch is phonemic in languages that use lexical tone, older adults’ struggles perceiving differences in dynamic pitch suggest they may have trouble learning to use such pitches phonemically.

Another difference between older and younger adults is in the role working memory plays in successful second language learning. Compared to younger adults, older adults’ success learning a second language depends more heavily on their baseline working memory capacity. Service and Craik (1993) demonstrated that compared to younger adults, older adults’ ability to learn new vocabulary items depended more on their ability to store the items in phonological working memory, suggesting that cognitive mechanisms interact differently with language learning as a function of age. Mackey and Sachs (2012) undertook a study of English question formation by native Spanish speakers aged 65 years or older. Working memory capacity was positively correlated with learners’ ability to form grammatical questions at post-test. Because Mackey and Sachs (2012) used similar methods to other studies investigating second-language question formation in younger adults (e.g., Mackey and Philp, 1998), they were able to compare their older adult data to younger adult data collected via similar paradigms. This comparison revealed that though both older and younger adults showed learning from pre- to post-test, only the younger adults showed evidence of sustained learning to a follow-up maintenance test.

Though these data refer to non-native semantic and syntax learning, a reasonable first hypothesis for investigating non-native speech learning is that older adults’ learning success will depend on working memory capacity though no such relationship has been seen in younger adults’ learning.

Beyond their reliance on working memory capacity to successfully learn non-native language items, older adults have deficits in category learning compared to younger adults (Maddox et al., 2010; Glass et al., 2012). Specifically, older adults show impairments in their ability to learn A/B categories (when an exemplar can be a member of one of two possible categories). Such category learning is indicative of the type of learning necessary for successful non-native speech perception, as listeners must categorize a speech token to one of several phonological categories. Further, when non-native phonological learning is combined with a lexical learning task, the listener must assign a stimulus token to one semantic category for perceptual success. Older adults’ difficulties with A/B category learning are thought to result from age-related declines in declarative memory capacity (Maddox et al., 2010; Glass et al., 2012), suggesting that older adults’ success learning non-native phonology in the context of lexical learning is related to declarative memory capacity (Ullman, 2001, 2004).

The present study tested older adults’ ability to learn non-native phonology, namely lexical tone, in a paradigm previously demonstrated to be successful in younger adults (Perrachione et al., 2011). Briefly, that paradigm built on Wong and Perrachione’s (2007) finding that listeners with a high baseline sensitivity for dynamic pitch showed more rapid learning and higher final levels of attainment for lexical tone than did listeners with low baseline sensitivity for dynamic pitch. Perrachione et al. (2011) hypothesized that listeners with low baseline sensitivity for dynamic pitch could be more successful learners if the amount of uninformative variation across stimuli was reduced, allowing the listener to focus on the pitch contrast and eliminating the need to integrate indexical information across talkers. They tested this possibility by setting up a 2 × 2 design in which the first factor was baseline sensitivity for dynamic pitch, split into high- or low-sensitivity listeners. The second factor referred to the number of talkers who produced the training stimuli. In one condition, stimuli produced by four talkers (two female) were randomly mixed across training trials, called multi-talker training; in the other condition, all stimuli were produced by a single talker, called single-talker training. Regardless of training condition, all listeners received equal amounts of exposure to the training tokens. Following training, listeners whose baseline sensitivity for dynamic pitch was poor were more successful learning lexical tone in a single-talker paradigm whereas those listeners whose baseline sensitivity for dynamic pitch was high benefitted more from a multi-talker paradigm; no effect of working memory capacity was found. Applying this 2 × 2 design to older adults allows for an investigation as to whether individual variation in older adults’ sensitivity for dynamic pitch interacts with training paradigm. Alternatively, cognitive factors may be more predictive of older adults’ learning success (Service and Craik, 1993; Mackey and Sachs, 2012).

Based on older adults’ difficulty perceiving dynamic pitch, we expected that older adults would show reduced dynamic pitch perception compared to younger adults. We further expected that working memory capacity would be a significant predictor of learning outcomes in older adults (e.g., Service and Craik, 1993; Mackey and Sachs, 2012). Though we expected older adults’ baseline dynamic pitch sensitivity to be low, we anticipated that there would be sufficient individual variation to see an interaction with the training paradigm such that those listeners with relatively high sensitivity would show a greater benefit from multi-talker training whereas those listeners with relatively low sensitivity would show a greater benefit from single-talker training (e.g., Perrachione et al., 2011). Finally, we assessed older adults’ declarative memory capacity with the expectation that declarative memory capacity would predict learning success (e.g., Maddox et al., 2010; Glass et al., 2012).

## Materials and Methods

### Participants

Forty-eight older adults (30 female) participated. Forty-three older adults (27 female) were recruited from the Northwestern University community and participated at Northwestern University; the remaining five participants (three female) were recruited from the Florida State University community and participated at Florida State University. All participants were older than 55 years, with a mean age of 65.06 years (*SD* = 6.78 years). Participants self-reported normal hearing and 40 listeners had hearing thresholds less than 25 dB HL at 0.5, 1, 2, 4, and 8 kHz as assessed by puretone audiometry; eight participants had hearing thresholds lower than 25 dB HL at 0.5 and 1 kHz but elevated thresholds at higher frequencies, though thresholds were below 40 dB HL at all frequencies. Participants self-reported no known cognitive deficits and scored 25 or greater on the Mini-Mental State Exam (Folstein et al., 1975). All participants were native English speakers and no participant had any previous experience with tone languages. This study was reviewed and approved by the Institutional Review Boards of Northwestern University and Florida State University. All participants gave written informed consent.

Listeners varied in their previous musical and language experience. Eighteen reported having some knowledge of a second language, but no participant reported being proficient in a language other than English. Thirty reported previous musical experience. The most common type of musical experience was private lessons on an instrument, with a mean duration of 5.7 years.

Participants were divided into High-Aptitude Learners or Low-Aptitude Learners based on their performance on the Pitch Contour Perception Test, discussed below [PCPT; (Perrachione et al., 2011)]. Previous research has used a criterion of 70% accuracy to divide listeners into high-aptitude listeners (HAL) vs. low-aptitude listeners (LAL) (Wong and Perrachione, 2007; Perrachione et al., 2011; Chandrasekaran et al., 2010; Ingvalson et al., 2013). However, given older adults’ difficulty identifying dynamic pitch (Shen et al., 2016), we adopted a criterion of 58% accuracy—significantly different from chance performance for a two-alternative forced-choice task—to separate the listeners into HAL and LAL groups. HAL listeners were significantly better than chance at identifying dynamic pitch (accuracy *M* = 67%, *SD* = 10%), LAL listeners showed chance performance (accuracy *M* = 51%, *SD* = 3%). Only 8 listeners of 48 (16.67%) who completed the PCPT were able to identify the dynamic pitch patterns at 70% accuracy, in contrast to the 31 of 64 younger adults (48.44%) assessed by Perrachione et al. (2011), further demonstrating the difficulty older adults have with dynamic pitch tasks. Twenty-four participants were placed into each group. Within each learning group, participants were randomly assigned to multi-talker training or single-talker training, for a 2 × 2 design with 12 listeners per cell. To ensure the HAL and LAL groups were matched on demographic variables, age, gender, years of musical training, and knowledge of another language were compared via *t*-tests; there were no significant differences between the groups (all *p* > 0.05).

### Materials

Participants’ ability to perceive pitch was assessed using the Pitch Contour Perception Test (PCPT). In this test, the vowels /a, e, i, o, u, y/ were produced by four native Mandarin speakers, two male and two female, using a high level tone (tone 1). Each vowel token was superimposed with level, rising, or falling pitch contours using the pitch-synchronous overlap and add (PSOLA) method in Praat. Pitch contours were based on each talker’s level tone production, creating natural variability across talkers. Talkers for the PCPT test were different from the talkers who produced the training stimuli, described below. Using different stimuli, different talkers, and a different task than the training—specifically, using a non-lexical task—allows us to test listeners’ baseline sensitivity to pitch patterns outside of lexical tone perception and lexical tone learning.

The lexical tone learning materials for the current study were the same as those in Perrachione et al. (2011). Pseudo-word stimuli created by Wong and Perrachione (2007) were used for training. Six syllables (dree, nuck, fute, pesh, nare, and vess) were each produced by eight native American English speakers (four female). English pseudowords were chosen because the main aim of the experiment was to examine how non-native English speakers, for whom pitch is not phonetically contrastive, learn to use pitch lexically. Stimuli that contain native phonological patterns are easier to learn than those that use non-native phonological patterns (Feldman and Healy, 1998). Syllables were superimposed with level, rising, and falling contours using the PSOLA method in Praat to create 18 pseudo-words that differed in both syllable and lexical tone (six syllables × three tones). The 18 pseudo-words were paired with 18 common objects (e.g., bus, cow, and hairbrush) for the word-learning portion of the experiment; the pseudo-words and their meanings can be seen in **Table 1**. Four talkers were designated for training (two female) whereas four talkers (two female) were reserved for generalization testing.

**Table 1:** Mandarin-like pseudo-words and meanings learned during training.

p^h^ɛ∫ 1	dɹi1	nɛɹ1	vɛs1	n∧k1	fjut1
(glass)	(arm)	(boat)	(hat)	(brush)	(shoe)
p^h^ɛ∫ 2	dɹi2	nɛɹ2	vɛs2	n∧k2	fjut2
(pencil)	(phone)	(potato)	(tape)	(tissue)	(book)
p^h^ɛ∫ 4	dɹi4	nɛɹ4	vɛs4	n∧k4	fjut4
(table)	(cow)	(dog)	(piano)	(bus)	(knife)

*Numbers following the lexical items designate tone. Level tone is indicated by 1, rising tone by 2, and falling tone by 4. During training, word meanings were represented pictorially.*

For both the isolated vowel and pseudo-word stimuli, resynthesis was applied across the sonorous segments of each token, using each talker’s mean F0 across all productions as a baseline value. The level contour began and ended at the baseline pitch; the rising contour began at 74% of baseline and ended at the baseline pitch; the falling contour began at 110% of baseline and fell by 45%. The resulting contours are in line with measurements obtained by Shih (1988), and the pitch contours have been shown to be perceptually natural to native Mandarin speakers (Wong and Perrachione, 2007). Onset and offset values were varied by ±3% across talkers to produce talker-specific variability in the target phonemic contrast.

### Cognitive Assessments

Working memory ability was conceptualized as the ability to briefly store and manipulate verbal information during processing (Baddeley and Hitch, 1974) and was assessed using the Woodcock Johnson Test of Cognitive Abilities (Woodcock et al., 2007) subtests on Non-word Repetition, and Auditory Working Memory. Phonological awareness was conceptualized as the ability to manipulate the sounds of oral language (e.g., Gillon, 2004) and was measured by the Woodcock–Johnson Test of Cognitive Abilities Sound Blending subtest. Declarative memory is often assessed via recall tasks, included cued-recall (Knowlton and Squire, 1995). We assessed declarative memory using the Wechsler Memory Scale (Wechsler, 1945) subtests on Logical Memory and Verbal Paired Associates. Both the Woodcock–Johnson and the Wechsler Memory Scale have been normed for older adults. Assessments were given and scored per standard procedures.

### Baseline Dynamic Pitch Assessment

When administering the PCPT, each tone-vowel token was repeated three times, for a total of 180 stimuli (five vowels × four talkers × three tones × three presentations). Listeners identified the pitch contour of a given stimulus by matching their auditory percept to arrows presented on a computer screen (→, ↗, or ↘) in a two-alternative forced-choice task. Only two alternatives were presented on a given trial; order of response alternatives was counterbalanced on each trial. Pilot testing indicated that older adults required a longer processing time than younger adults for this task, and the inter stimulus interval (ISI) was increased to 1500 ms (Wong and Perrachione, 2007). Longer ISIs have been shown to be more beneficial for older than younger adults (Meijer et al., 2006), thought to be due to reduced speed of processing in older adults. Performance on the PCPT has been linked to Mandarin lexical tone learning performance in younger adults (Wong and Perrachione, 2007; Chandrasekaran et al., 2010; Perrachione et al., 2011; Ingvalson et al., 2013).

### Procedure

Prior to training, all participants completed the MMSE, the subtests of the Woodcock–Johnson Tests of Cognitive Abilities, and the PCPT.

Within each aptitude grouping, listeners were randomly assigned to either multi-talker or single-talker training. The training paradigm was identical for the two training types, the only difference between the two was the number of talkers the listeners heard. Multi-talker training used all four of the talkers selected for training; all stimuli were produced by all talkers in each training session with random trial-by-trial variation in talkers. Single-talker training presented a single talker throughout all sessions; talkers were counterbalanced by subject.

Over a triad of trials, listeners heard three syllables that differed only in pitch contour. The picture that indicated the pseudo-word’s meaning appeared in conjunction with the pseudo-word. After four presentations of each pairing, listeners were asked to identify the meaning of a given pseudo-word from the trained options (three-alternative forced-choice). A 3AFC task was used for lexical training to highlight the pitch differences among syllables. Feedback was given on each trial, and the correct response was presented if an incorrect response was given. Thus, listeners learned the pseudo-words as minimal triplets that differed only in lexical tone, though stimuli were presented in isolation. On each training day, listeners heard 72 total tokens (18 pseudo-words × 4 presentations). In the multi-talker training type, each pseudo-word token was spoken by a different talker; in the single-talker training type, each pseudo-word token was spoken four times. The training session ended with an assessment of the day’s learning. In the final assessment, all 72 tokens were randomly intermixed in an 18-alternative forced-choice task. No feedback was given. For the multi-talker training type, all talkers were randomly intermixed in the learning assessment; in the single-talker training type, four tokens of each pseudo-word were presented in the final assessment, all spoken by the same talker. All listeners completed 8 days of training (Perrachione et al., 2011). Listeners were permitted to miss up to, but not more than, 2 days between any two given training sessions (e.g., training could be missed over the weekend) and not more than 2 days could pass between the final training session and the post-test.

After completing the 8 days of training, listeners returned on a separate day to complete the Test of Learning Achievement (TLA; Perrachione et al., 2011). This test was very similar to the final identification test used during the training component, but it used the four talkers reserved for generalization. As before, listeners heard one pseudo word—this time always spoken by an unfamiliar talker—and identified its lexical meaning from the complete list of 18 items. Each stimulus was presented only once and no feedback was given.

## Results

### Cognitive Differences between Aptitude Groupings

We entered the PCPT, the standardized scores from the three Woodcock–Johnson subtests, and the standardized scores from the two Wechsler Memory Scale subtests into six 2 × 2 (group × training) ANOVAs to determine if there were any differences between the HAL and LAL groups or between the multi- and single-talker training conditions on these variables. As anticipated, the HAL group had significantly higher performance on the PCPT than the LAL group *F*(1,44) = 48.24, *p* < 0.001. Additionally, the HAL group had higher performance than the LAL group on all but one of the cognitive assessments: the Sound Blending, Numbers Reversed, and Auditory Working Memory subtests of the Woodcock–Johnson Test of Cognitive Abilities, and the Logical Memory subtest of the Wechsler Memory Scale. The two groups were equivalent only on the Verbal Paired Associates subtest of the Wechsler Memory Scale. Conversely, the multi- and single-talker conditions differed only on the Verbal Paired Associates subtests of the Wechsler Memory Scale, but no other demographic measures. No interactions were significant. All means and standard deviations are shown in **Table 2**, both as a function of group (HAL or LAL) and training type (multi- or single-talker). The sources tables from the ANOVAs are shown in **Table 3**.

**Table 2:** Means of standardized scores, standard deviations, *F*-statistics, and *p*-values for the perceptual and cognitive assessments as a function of aptitude grouping and training type.

High- vs. Low-Aptitude Listeners						
	High		Low			
Assessment	*M*	*SD*	*M*	*SD*	*F*	*p*
Pitch Contour Perception Test	1.93	0.23	1.59	0.06	48.24	0.00
Sound Blending	118.17	10.87	106.38	13.95	10.91	0.00
Numbers Reversed	108.75	15.08	98.50	17.65	4.52	0.04
Auditory Working Memory	117.42	11.16	109.21	12.92	5.86	0.02
Logical Memory	11.83	2.51	9.67	3.23	6.79	0.01
Verbal Paired Associates	10.91	2.87	10.13	4.06	0.65	0.42

**Multi- vs. Single-Talker Training**						

	**Multi-Talker**		**Single-Talker**			
**Assessment**	***M***	***SD***	***M***	***SD***	***F***	***p***

Pitch Contour Perception Test	1.77	0.25	1.75	0.22	0.06	0.81
Sound Blending	115.29	13.10	109.25	13.94	2.86	0.10
Numbers Reversed	105.04	18.44	102.21	15.80	0.35	0.56
Auditory Working Memory	116.83	12.01	109.79	12.52	3.95	0.05
Logical Memory	11.38	2.93	10.13	3.13	2.26	0.14
Verbal Paired Associates	11.79	3.22	9.17	3.37	7.12	0.01

**Table 3:** Results from the ANOVAs from the perceptual and cognitive assessments as a function of aptitude grouping and training type.

Pitch Contour Perception Test					
Factor	*SS*	*df*	*MS*	*F*	*p*
Aptitude Grouping	1.37	1	1.37	48.24	0.00
Training Type	2.00E-03	1	2.00E-03	0.06	0.81
Group × Training	0.03	1	0.03	1.20	0.28
Residuals	1.25	44	0.03		

**Sound Blending**					

Aptitude Grouping	1669.00	1	1668.50	10.91	0.00
Training Type	438.00	1	438.00	2.86	0.10
Group × Training	23.00	1	22.70	0.15	0.70
Residuals	6728.00	44	152.90		

**Numbers Reversed**					

Aptitude Grouping	1261.00	1	1260.80	4.52	0.04
Training Type	96.00	1	96.30	0.35	0.56
Group × Training	21.00	1	21.30	0.08	0.78
Residuals	12283.00	44	279.20		

**Auditory Working Memory**					

Aptitude Grouping	809.00	1	808.50	5.86	0.02
Training Type	595.00	1	595.00	4.31	0.04
Group × Training	42.00	1	42.20	0.31	0.58
Residuals	6071.00	44	138.00		

**Logical Memory**					

Aptitude Grouping	56.30	1	56.33	6.79	0.01
Training Type	18.70	1	18.75	2.26	0.14
Group × Training	0.70	1	0.75	0.09	0.77
Residuals	365.20	44	8.30		

**Verbal Paired Associates**					

Aptitude Grouping	7.30	1	7.29	0.65	0.42
Training Type	79.50	1	79.47	7.12	0.01
Group × Training	1.20	1	1.22	0.11	0.74
Residuals	479.80	44	11.16		

### Dynamic Pitch Perception for Older and Younger Adults

To test our hypothesis that older adults would have lower dynamic pitch perception than younger adults, we compared the mean performance of the older adults in the present study to the mean performance of the younger adults in Perrachione et al. (2011). Perrachione et al. (2011) reported *M* = 84%, *SD* = 8% on the PCPT for the HAL group and *M* = 58%, *SD* = 8% on the PCPT for the LAL group. Older adults in the present study scored *M* = 67%, *SD* = 10% for the HAL group, whereas LAL listeners showed chance performance *M* = 51%, *SD* = 3%. Thus, the older adults in the HAL group scored 2.12 SD below the younger adult mean on the PCPT; older adults in the LAL group scored 0.88 SD below the younger adult mean on the PCPT.

### Training

**FIGURE 1:**
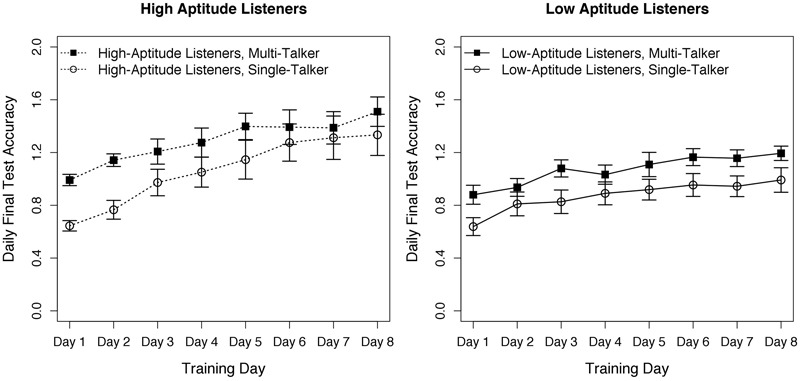
**Progress (arcsine transformed proportion correct) across the eight training days as a function of group (High- vs. Low-Aptitude Listeners) and training type (multi- vs. single-talker training).** Error bars are standard error of the mean. In the arcsine transform, floor performance is 0.12, chance performance is 1.57 and perfect performance is 3.02. The HAL group showed steeper learning than the LAL group, but there was no effect of training type.

Before analysis, all data were arcsine transformed (Studebaker, 1985) to mitigate floor effects in older adults’ performance. In line with Perrachione et al. (2011) learning rate was determined for each listener as the linear slope between pre-training (chance) and training session four (training midpoint); training data are shown in **Figure 1**. Slope values were submitted to a 2 × 2 (group × training) between-subjects ANOVA. There was a main effect of group, *F*(1,44) = 4.52, *p* = 0.04. The HAL listeners had steeper learning than did the LAL listeners across training conditions. However, there was no impact of training condition, *F*(1,44) = 0.90, *p* = 0.35, nor a significant interaction, *F*(1,44) = 0.005, *p* = 0.94.

A correlation matrix indicated that several variables were moderately correlated. Sound blending score was correlated with the PCPT subtest score, *r*(46) = 0.43, *p* = 0.002. Auditory Working Memory subtest score was correlated with the Numbers reversed subtest score, *r*(46) = 0.54, *p* < 0.001, the Logical Memory subtest score, *r*(46) = 0.54, *p* < 0.001, and the Verbal Paired Associates subtest score, *r*(45) = 0.40, *p* = 0.005. The relationship between PCPT score and TAL outcomes was one of our primary research questions. We therefore opted to remove the Sound Blending subtest score from subsequent analyses rather than combine it with PCPT score. This allowed us to assess the relationship between PCPT score and TAL performance without multicollinearity between our measure of interest and other cognitive assessments. Because the Auditory Working Memory subtest score was correlated with both another measure of working memory and with both measures of declarative memory, we opted to omit it from the subsequent analysis, as it appeared it was tapping both working and declarative memory. Finally, the two measures of declarative memory, the Logical Memory and Verbal Paired Associates subtests, were correlated, *r*(45) = 0.52, *p* < 0.001, and these were therefore combined into a single unified measure of declarative memory. The remaining measures were PCPT score, Numbers Reversed subtest, and a composite measure of declarative memory. These measures assessed listeners’ sensitivity for dynamic pitch, auditory working memory, and declarative memory. The HAL and LAL groups did not significantly differ on this composite measure of declarative memory, *t*(46) = 1.85, *p* = 0.07, but the two training groups did, *t*(46) = 2.38, *p* = 0.02.

To test our hypothesis that declarative and working memory capacity would predict TLA performance, we created an omnibus linear regression model. Independent variables were PCPT score, standardized Numbers Reversed score from the Woodcock–Johnson, and the unified measure of declarative memory (total of three predictive factors); the dependent variable was TLA performance. All model factors were entered at once; model factors and their contributions are presented in **Table 4**. This model demonstrated that only the declarative memory composite significantly predicted TLA performance for the older adults, *b* = 0.053, *t*(44) = 2.89, *p* = 0.01. The overall model was a significant fit, *R*^2^ = 0.32, *F*(36,44) = 6.86, *p* < 0.001.

**Table 4:** Predictors of TLA performance from an omnibus linear regression model.

	*b*	*SE*	*t(44)*	*p*
PCPT	0.18	0.22	0.84	0.40
Numbers Reversed	5.06E-03	3.16E3-03	1.60	0.11
Declarative Memory Composite	0.05	0.02	2.88	0.01

To test our hypothesis that training condition would interact with dynamic pitch sensitivity to impact TLA performance, we tested whether adding training group to the above regression model would account for additional variance (Perrachione et al., 2011). We created a second regression model that included training condition, PCPT score, Numbers Reversed score, the declarative memory composite, and interactions between training condition and PCPT score, between training condition and Numbers Reversed score, and training condition and declarative memory composite. Adding training group as a covariate did not significantly improve model fit, *F*(44,40) = 1.29, *p* = 0.29.

### Tone vs. Segmental Errors

Our final analysis was an investigation of the older adults’ error patterns. For all listeners, we computed the proportion of the total errors made in the last training session that were tone errors (errors made by misidentifying the pitch pattern) vs. those that were segmental errors (errors made by misidentifying the syllable; errors made for both tone and segment were counted in both categories). High proportions of tone errors indicate that listeners have learned the segments (Wong and Perrachione, 2007). Accuracy rates on the TLA correlated with the proportion of tone errors in the final training session, *r*(46) = 0.55, *p* < 0.001, indicating that listeners who had mastered the segments had better performance at the final test. Proportion of tone errors on the final training day were highly correlated with proportion of tone errors on the TLA, *r*(45) = 0.83, *p* < 0.001. To test the extent to which dynamic pitch perception and cognitive factors predicted error rates on the TLA we constructed an ordinary least-squares regression model with the proportion of tone errors on the TLA as the dependent variable and PCPT score, Numbers Reversed standardized score, and declarative memory composite as factors. We used the proportion of tone errors on the TLA instead of at the final training session based on the high correlation between tone error rates on the TLA and the final training session and because all listeners completed identical versions of the TLA but final training sessions were not identical for all listeners (listeners in the multi-talker condition heard four talkers intermixed, but listeners in the single-talker condition only heard one talker). All factors were entered into the model at once; model factors and their contributions are shown in **Table 5**. The declarative memory composite was a significant predictor of tone error rates, *b* = 0.03, *t*(43) = 3.04, *p* = 0.004 and the overall model was a significant fit, *R*^2^ = 0.28, *F*(3,43) = 5.62, *p* = 0.002.

**Table 5:** Predictors of the proportion of tone errors in the final training session from an omnibus linear regression model.

	*b*	*SE*	*t(44)*	*p*
PCPT	-0.16	0.10	-1.55	0.13
Numbers Reversed	2.40E-03	1.50E-03	1.60	0.12
Declarative Memory Composite	0.03	0.01	3.04	0.004

**FIGURE 2:**
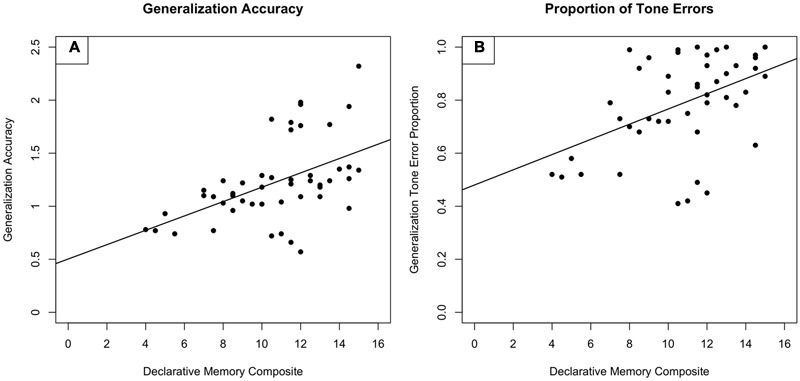
**Performance on (A)** the Test of Learning Achievement as predicted by a declarative memory composite score and **(B)** the rate of tone errors on the Test of Learning Achievement as predicted by a declarative memory composite score. The declarative memory composite score included the Logical Memory and Verbal Paired Associates subtests of the Wechsler Memory Scale.

Across these analyses, declarative memory capacity appears to be predictive of TLA performance. One interesting component of these findings is that though the HAL group scored more highly on most cognitive measures, our composite measure of declarative memory differed on the basis of training condition, not aptitude group. Thus, though baseline dynamic pitch sensitivity is confounded with many of the cognitive measures, it is not confounded with declarative memory. This in turn suggests that declarative memory capacity is predictive of TLA performance for older adults. Relationships between declarative memory and TLA performance, and between declarative memory and tone error rate are shown in **Figure 2**.

We hypothesized that older adults’ TLA performance would be predicted by working memory and/or declarative memory performance. We further hypothesized that those older adults who had relatively high baseline pitch sensitivity—indicated by higher performance on the Pitch Contour Perception Test (PCPT)—would show greater performance on the TLA following multi-talker training whereas those older adults who had relatively low baseline pitch sensitivity would show greater performance on the TLA following single-talker training (Perrachione et al., 2011). Regression analyses indicated that baseline aptitude for dynamic pitch was not associated with TLA performance, nor did it interact with training paradigm. Rather, declarative memory was predictive of older adults’ performance.

## Discussion

The aim of the current study was to investigate older adults’ ability to learn lexical tone in the context of a paradigm previously shown to be successful for optimizing individual differences in younger adults’ learning outcomes. We expected working memory capacity and declarative memory capacity to be associated with of older adults’ generalization performance, though earlier work showed younger adults’ learning was not associated with working memory capacity. We further expected older adults’ generalization performance to be associated with an interaction of baseline dynamic pitch sensitivity and training paradigm.

These expectations were only partially realized. High-aptitude older adults’ baseline sensitivity to dynamic pitch was lower than high-aptitude younger adults, but both low-aptitude younger adults and low-aptitude older adults performed at chance level. The lower performance by high-aptitude older adults meant that high-aptitude younger adults were 3 SD above chance performance while high-aptitude older adults were closer to chance. Possibly due to this reduced variation, dynamic pitch sensitivity was not associated with final learning outcomes for older adults, though it had been shown to be highly linked to learning outcomes for younger adults (Wong and Perrachione, 2007; Perrachione et al., 2011). Working memory capacity also was not associated with generalization performance but declarative memory capacity was.

As described in the section “Introduction,” older adults are poorer than younger adults at A/B category learning. This is thought to stem from age-related declines in declarative memory capacity (Maddox et al., 2010; Glass et al., 2012). Because the current study trained lexical tone in the context of learning to associate meaning with tone-syllable pairings, the listeners’ task was analogous to an A/B categorization task in that for each auditory token, the listener had to choose the correct meaning. Particularly for the TLA, listeners were required to utilize rules for mapping phonetics to semantics with novel category exemplars. Given that this sort of learning is associated with declarative memory in older adults, it is therefore not surprising that those older adults with relatively larger declarative memory capacity showed better performance on the TLA. The fact that older adults’ error types in the TLA was also predicted by declarative memory capacity further demonstrates that those listeners with larger declarative memory capacity were better able to learn the categorization rules in that they were less likely to mis-categorize based on the easily perceived segmental information. Of ongoing interest is the relationship between training type and declarative memory. In this study, declarative memory was assessed post-training and the two training groups differed significantly in their declarative memory capacity. Future studies should assess declarative memory pre-training to ensure all training groups are matched in declarative memory capacity pre-training. Post-test assessment of declarative memory will indicate if training type leads to gains on declarative memory assessments and whether declarative memory capacity interacts with training type.

The lack of relationship between PCPT score and generalization performance highlights older adults’ struggles identifying pitch patterns and using pitch patterns to identify lexical items. Ingvalson et al. (2013; see also Cooper and Wang, 2013) trained younger adults’ with poor baseline sensitivity for pitch patterns to identify pitch patterns prior to introducing lexical training. Those listeners who received the pre-training in pitch identification better learned the lexical categories than those listeners who received the lexical-only training similar to the training used here. One possibility for improving older adults’ lexical tone learning, then, may be to focus first on improving their ability to differentiate pitch patterns before moving on to training lexical tone. Another possibility recognizes that eight of our participants had mild hearing loss (between 25 and 40 dB HL) at 2, 4, and 8 kHz. When the falling pitch, tone four, is spoken by a female talker, the peak of the tone was around 2700 Hz in the /i/ and /y/ vowels used in the PCPT test, and around 1800 Hz in the training and testing stimuli. Though all stimuli were presented at 70 dB SPL, there is the possibility that the higher frequencies were difficult to perceive. Post-training questionnaires indicated that listeners did not have difficulty perceiving the pitch patterns but instead struggled to remember the mapping between pitch and meaning, supporting the interpretation that a declarative memory strategy drove generalization performance. Nonetheless, the possibility remains that the older adults had difficulty perceiving the lexical tones but were not aware of it. Future efforts could attempt exaggerating the pitch patterns which might make the phonetic distinctions more salient and/or more audible, though this change would eliminate the naturalness of the lexical tone movements present in the stimuli (Wong and Perrachione, 2007), meaning listeners would need to learn to perceive pitch patterns consistent with those produced by native talkers in order to show evidence of phonetic learning (McCandliss et al., 2002).

Another possibility to improve older adults’ lexical learning may be to reduce reliance on declarative memory, which is an area of weakness in older adults. Investigations into speech category learning in younger adults have demonstrated that listeners are more successful when they employ an information-integration strategy supported by procedural memory than when they use a rule-based strategy supported by declarative memory (Chandrasekaran et al., 2014). Listeners who were encouraged to attend to the non-preferred dimension showed a more rapid transition toward information-integration strategies; in the case of lexical tone, native English listeners overweight pitch height relative to pitch direction (Chandrasekaran et al., 2007) and were therefore encouraged to attend to pitch direction. Older adults are less willing to use an information-integration strategy for non-native speech learning than younger adults (Chandrasekaran et al., 2014) and may therefore benefit from explicit instruction to encourage procedural memory-based learning and thereby reduce the importance of declarative memory capacity. Encouraging older adults to place greater weight on pitch direction could also potentially aid in their perception of dynamic pitch, though of course this possibility needs to be explicitly tested.

These data demonstrate that older adults’ difficulties perceiving dynamic pitch impacts their ability to learn to use pitch phonemically. One weakness of our study is that it lacks a direct comparison to younger adult data, though we followed the methods that Perrachione et al. (2011) used in younger adults. Whether current paradigms can be adapted to accommodate older adults’ challenges to learning remains to be seen, and these efforts will benefit from direct comparisons between younger and older adults. We look forward to seeing future efforts to elucidate the mechanisms of non-native speech learning in older adults, as well as efforts to improve older adults’ speech learning outcomes.

## Ethics Statement

This study was carried out in accordance with the recommendations of the Institutional Review Boards of Northwestern University and Florida State University. All participants gave written informed consent in accordance with the Declaration of Helsinki.

## Author Contributions

EI and PW developed the research. CN and AZ collected the data. EI analyzed the data. EI and PW wrote the manuscript.

## Conflict of Interest Statement

The authors declare that the research was conducted in the absence of any commercial or financial relationships that could be construed as a potential conflict of interest.
